# Loss of the flagellar regulator FlhC changes the transcriptional response of plant-associated *Acidovorax delafieldii* strains to metabolites from *Rhizophagus irregularis*-colonized *Lotus japonicus* roots

**DOI:** 10.1093/ismeco/ycaf235

**Published:** 2026-01-19

**Authors:** Roberto Siani, Yang Si, Stefanie Katharina Thaqi, Georg Stabl, Caroline Gutjahr, Michael Schloter

**Affiliations:** Technical University of Munich, TUM School of Life Sciences, Chair of Environmental Microbiology, Munich, Germany; Research Unit for Comparative Microbiome Analysis, Helmholtz Munich, Munich, Germany; Technical University of Munich, TUM School of Life Sciences, Department of Plant Genetics, Munich, Germany; Department of Root Biology and Symbiosis, Max Planck Institute of Molecular Plant Physiology, Potsdam-Golm, Germany; Technical University of Munich, TUM School of Life Sciences, Chair of Environmental Microbiology, Munich, Germany; Research Unit for Comparative Microbiome Analysis, Helmholtz Munich, Munich, Germany; Technical University of Munich, TUM School of Life Sciences, Department of Plant Genetics, Munich, Germany; Department of Root Biology and Symbiosis, Max Planck Institute of Molecular Plant Physiology, Potsdam-Golm, Germany; Technical University of Munich, TUM School of Life Sciences, Department of Plant Genetics, Munich, Germany; Department of Root Biology and Symbiosis, Max Planck Institute of Molecular Plant Physiology, Potsdam-Golm, Germany; Technical University of Munich, TUM School of Life Sciences, Chair of Environmental Microbiology, Munich, Germany; Research Unit for Comparative Microbiome Analysis, Helmholtz Munich, Munich, Germany

**Keywords:** plant-microbe interactions, *Acidovorax*, flagellar regulation, mycorrhiza, plant microbiome, transcriptional regulation

## Abstract

*Acidovorax* is a cosmopolitan bacterial genus comprising both beneficial and pathogenic plant-associated species. In plant-associated bacteria, flagella can facilitate colonization but also trigger plant immune responses driving mutation or loss of flagella in some strains. FlhC is an important transcriptional regulator of flagella assembly genes. Here, we investigated whether it modulates root colonization and the transcriptional response of plant-commensal *Acidovorax* strains to different plant cues. As model cues, we used root extracts from *Lotus japonicus* that forms symbioses with arbuscular mycorrhiza fungi (AMF), which strongly change root metabolism and add their own metabolites. To offer different stimuli to *Acidovorax* bacteria, we used extracts from *L. japonicus* roots colonized or not with the AMF *Rhizophagus irregularis*. We investigated two *Acidovorax delafieldii* strains with almost identical genomes but differences in the presence or absence of *flhC*. Overall, loss of *flhC* resulted in distinct expression profiles and a more modular transcriptional network. Pathway analysis linked *flhC* to genes associated with stress tolerance, nutrient mobilization, biofilm formation, secretion, surface attachment, and quorum sensing. Root extracts from mycorrhizal roots induced increased expression of genes associated with substrate preference and downregulation of genes involved in biofilm formation and secretion. *flhC*-deficient strains also responded with increased expression of genes related to surface attachment and vitamin biosynthesis. The absence of *flhC* correlated with increased root colonization and influenced the response of *Acidovorax* to *R. irregularis in planta*. Our findings highlight that a flagellar regulator in root-associated bacteria influences root colonization and transcriptional responses to host cues.

## Introduction

Bacterial associations with plant roots can result in mutually beneficial, or pathogenic, or neutral commensal relationships partly regulated by the plant host [1, 2]. To minimize the risk of pathogenic infections, the plant’s immune system distinguishes harmful microbes [3]. As part of the plant immune response, specialized receptors can detect microbe associated molecular patterns (MAMPs), such as peptidoglycans and flagellins of the bacterial cell wall and flagellum [4]. In fact, the thousands of flagellin proteins constituting the filament of the bacterial flagellum are highly immunogenic [5]. Flagella are motility organelles widespread in phylogenetically diverse bacterial taxa, found in both pathogenic and non-pathogenic species. Pathogenic bacteria that depend on flagella for infection often evolve mechanisms to evade detection while retaining motility [6]. Alternatively, bacteria for which motility is less advantageous may eventually lose their flagella entirely, which results in populations with distinct behaviour [7–9]. Flagellar assembly is controlled by a hierarchical gene system, with regulators at each level activating the transcription of the next class of genes. The class I genes *flhC* and *flhD* encode the components of the FlhD₄C₂ complex (hereafter referred to as FlhDC), which binds to the promoters of class II genes to initiate flagellar construction [10]. Consequently, mutations in *flhDC* can disrupt flagellar assembly entirely [11]. To coordinate motility with other cellular processes, FlhDC is thought to influence a range of traits, including virulence, metabolism, and growth, in many bacterial species [12, 13]. As an example, in plant pathogens of the genus *Dickeia*, FlhDC co-regulate the secretion of cell wall degrading enzymes [14, 15]. However, FlhDC regulatory interactions with non-flagellar genes have been mostly investigated in the context of enterogastric bacteria such as *Salmonella* [16], *Escherichia* [1, 7], and *Yersinia* [17]. Most studies on plant-associated microbes focus on pathogens or a few well-known mutualists like nitrogen-fixing bacteria and plant growth–promoting rhizobacteria [14, 18]. In contrast, less is known about how root-associated lifestyles are regulated in bacteria with unclear ecological roles, such as neutral commensals. As these bacteria are increasingly recognized as relevant to plant health [19, 20], studying key regulators like FlhC and their responses to plant signals can clarify their contribution to plant fitness.

The genus *Acidovorax* illustrates the diversity of plant-associated microbes, including well-known pathogens, such as *A. avenae* and *Acidovorax citrulli*, and beneficial or commensal root endophytes like *A. radices* and *Acidovorax delafieldii*. While many colonization factors have been identified in plant-associated *Acidovorax* strains [21], flagella are found in both pathogenic and non-pathogenic species. Genomic data indicate that these species encode homologs of the canonical *flhDC* master regulator of flagellar biogenesis [21, 22]. Two plant-commensal *A. delafieldii* strains LR124 and LR140 share highly similar genomes and differ by only two genes, one of which is *flhC*, which is absent from the LR124 genome. Both strains were initially isolated from healthy *L. japonicus* (ecotype Gifu) plants [23], a model legume commonly used to study plant-microbe interactions with nitrogen fixing rhizobia and arbuscular mycorrhizal fungi (AMF) [24–26]. In previous experiments, LR140 and LR124 had contrasting effects on *L. japonicus* root growth: LR140 had an adverse effect, whereas LR124 promoted growth [21], suggesting that the presence of flagella may have an influence on plant responses to the bacteria. Considering that flagellar motility represents a major strategical difference, that, in some pathogenic species, is associated with different stages of the infection cycle [27, 28], we investigated how and to what extent the loss of *flhC* reshapes the phenotype of plant-commensal *Acidovorax*. Plant roots contain many primary and secondary metabolites and their concentration and composition changes in response to abiotic and biotic cues. For example, symbiosis with arbuscular mycorrhiza fungi (AMF) influences the root metabolome [29] and affects a substantial portion of detectable root metabolites including the production of a wide range of putatively bioactive polyphenols [30]. Besides, fungal effector proteins have been shown to influence both plants and their microbiome composition [31]. We hypothesized that these metabolic shifts are sensed by bacteria and influence the bacterial transcript regulation [32] and its regulatory hubs, thus allowing identifying the role of *flhC* in response to host cues and co-occuring microbes.

To address these hypotheses, we adopted a reductionist approach, circumventing both feedback effects from host–microbe interactions and methodological constraints of *in planta* systems that arise from the stark differences in size and abundance between eukaryotic and prokaryotic cells, affecting the relative availability of bacterial RNA in transcriptomic datasets [33]. Thus, we grew *Acidovorax* strains in minimal medium supplemented with root extracts from *Lotus japonicus* roots colonized or non-colonized by the AMF *Rizophagus irregularis*. We also generated two LR140-derived mutants, a *flhC* deletion mutant (LR140*^ΔflhC^*) and its complemented version (LR140*^ΔflhC;flhC^*), to validate our observations and confirm that the transcriptional changes were specifically linked to *flhC*. With the help of the specialized database PLaBAse [34], we focused our analyses of transcriptional changes affecting genes associated with plant-microbiome interactions and/or plant growth promotion.

## Material and methods

### Strain selection and genome annotation

Two *A. delafieldii* strains, LR124 and LR140, were selected from 52 endophytic and commensal *Acidovorax* isolates obtained from healthy *L. japonicus* roots. Details on their isolation, sequencing, and the broader strain collection, including effects on plant growth, have been described previously [21, 23]. Genome assemblies are publicly available under accession number PRJEB37696 at the European Nucleotide Archive. Average nucleotide identity (ANI) between strains was calculated using fastANI v1.3 [35]. For this study, genome re-annotation was performed using Bakta v1.8.1 [36], InterProScan v5.64–96 [37] with Pfam 36.0 [38] and PLaBAse v1.02 [34]. PLaBAse enables the annotation of bacterial genes based on a curated set of traits relevant to the plant microbiome and employs an ontology structured across seven hierarchical levels (PGP0 to PGP6). This ontology facilitates the interpretation of transcriptional changes by grouping genes into functional categories and pathways (see examples in Table S1). The broadest distinction (PGP1) separates bacterial traits affecting the plant directly (e.g. secretion of effectors) from traits with potential indirect effects (e.g. microbe-microbe competition), with increasing specificity at each subsequent level. For example, PGP2 includes categories such as colonization, competition, remediation, and fertilization, while PGP5 comprises pathway-specific terms like phosphate solubilization or type II secretion. Reciprocal best hits between genomes were identified using MMseqs2 easy-rbh v13.45111 [39]. Annotations and ortholog mappings are provided in Supplementary Data S1.

### Construction of *flhC* in-frame deletion and complementation

*Acidovorax* LR140 was used as the parental strain to generate an in-frame deletion of *flhC* following a previously described protocol [40]. Briefly, upstream and downstream flanking regions of *flhC* were amplified using primers listed in Table S2A and cloned into the suicide vector pK19mobsacB via Golden Gate cloning. The resulting construct, pK19-*ΔflhC*, was introduced into *Escherichia coli* ST18, which served as the donor in a biparental conjugation with *A. delafieldii* LR140 as the recipient. Donor and recipient cells were mixed at a 1:3 ratio (based on OD) and plated on 50% TSB agar. After 24 h of incubation at 30°C, colonies were selected on kanamycin-treated TSB plates. Colonies were subsequently plated on 50% TSB agar supplemented with 10% sucrose to isolate double-crossover recombinants. Colonies that grew on TSB but not on kanamycin-containing plates were further verified by sequencing to confirm deletion of *flhC*. For complementation, the *flhC* coding region along with its native promoter was amplified using primers listed in Table S2B and cloned into plasmid pUC57 via Golden Gate cloning. The resulting plasmid was introduced into the *ΔflhC* mutant by biparental conjugation.

### Swimming motility assay

*Acidovorax* strains LR140, and *LR140*^*ΔflhC*;*flhC*^ (*flhC*+), LR124 and *LR140^ΔflhC^* (*flhC*−) were cultured in Tryptic Soy Broth (TSB) medium, and the optical density at 600 nm (OD_600_) of each culture was adjusted to 2.0. Ten microliters of each bacterial suspension were inoculated at the centre of soft TSB plates (0.3% agar) for 72 h at 21°C. Colony diameters were measured to assess motility. Four biological replicates were used for each strain.

### Cultivation of *L. japonicus* with *R. irregularis*

*L. japonicus* seeds were prepared by scarification with sandpaper, followed by surface sterilization in a solution of 10% DanKlorix Original (CP GABA GmbH, Germany) and 0.1% sodium dodecyl sulphate for 15 minutes. Sterilized seeds were washed five times in sterile distilled water and germinated on 0.8% water-agar plates, incubated for 3 days at 22°C in the dark and then for 4 days at 22°C under light (210 μmol m^−2^ s^−1^; growth cabinet PK520-LED, Polyklima GmbH, Germany). Seven-day-old plantlets were transferred to 7 × 7 × 8 cm Göttinger Pots (Hermann Meyer KG, Germany) filled with washed and autoclaved quartz sand (Casafino Quarzsand, fire-dried, 0.7–1.2 mm; BayWa AG, Germany). Five plants were grown per pot, with five pots per condition. A layer of synthetic cotton was placed at the bottom of each pot to prevent sand loss. Half of the pots (15 in total) were inoculated with 500 spores of *R. irregularis* (C-grade spores; Agronutrition SAS, France) for mycorrhizal treatment. All plants were maintained under controlled conditions: 60% relative humidity, light intensity of 150 μmol m^−2^ s^−1^, a 16 h light / 8 h dark cycle, and a temperature regime of 24°C (day) / 22°C (night). ‘Plants were watered three times per week, twice with sterile water and once with half-strength Hoagland’s solution [30]. After 8 weeks, roots were harvested. Most of them were freeze-dried for metabolite extraction. Two to three root systems per pot were separately processed to confirm *R. irregularis* colonization (>90%). After harvesting roots were incubated in 10% KOH at 95°C for 15 minutes, then in 10% acetic acid, followed by ink staining and de-staining with 5% acetic acid, as described previously [41]. Root fragments (1 cm) were mounted on microscopy slides and observed under 10× magnification using a light microscope. Arbuscular mycorrhizal colonization was quantified using a modified gridline intersect method [42].

### Spiked media preparation

Following the method of Nour et al. [43], crude extracts were prepared from control (*L. japonicus, Lj*) and *R. irregularis*–colonized *L. japonicus* (*Lj + Ri*). Ten grams of freeze-dried root biomass from each treatment were homogenized separately in 300 ml of distilled water using a tabletop blender. The homogenates were sequentially filtered through Miracloth, Whatman filter paper, and a sterile 0.22 μm syringe filter to remove plant debris and contaminants. The filtered extracts were then diluted 1:3 with M9 minimal medium (Merck KGaA, Darmstadt, Germany), prepared according to the manufacturer’s instructions. The resulting media are hereafter referred to as *Lj* (from uncolonized roots) and *Lj + Ri* (from mycorrhizal roots).

### Cultivation conditions

Actively growing colonies of LR124, LR140, LR140*^ΔflhC^*, and LR140*^ΔflhC;flhC^* were picked from R2A agar plates and used to inoculate 4 ml of *Lj* media. Cultures were incubated in a shaking incubator at 30°C and 124 rpm. Optical density was measured and recorded automatically over 48 hours to identify the onset of the stationary phase. After 24 hours, 10 μl of each culture were plated to confirm purity, and the remaining cultures were diluted to an OD^600^ of 0.4. Five biological replicates were prepared for each strain-media type combination by inoculating 100 μl of the diluted culture into 10 ml aliquots of *Lj* or *Lj + Ri* media. These cultures were incubated under the same shaking conditions (30°C, 124 rpm) for 24 hours. Cells were harvested by centrifugation at 3750 × g for 5 minutes, the supernatant was removed completely, and the resulting cell pellets were flash-frozen in liquid nitrogen and stored at −80°C until RNA extraction.

### RNA extraction and purification

Total RNA was extracted using the PureLink RNA Mini Kit (Invitrogen) with slight modifications to the manufacturer’s protocol. After a 5-minute lysozyme digestion for cell lysis, samples were homogenized in bead tubes using the Precellys24 Instrument (Bertin Technologies) at maximum speed for over 45 seconds. Column digestion steps were omitted. Following extraction, genomic DNA was removed using the RNA Clean & Concentrator-5 Kit (ZYMO Research). Absence of DNA contamination was confirmed by 16S rDNA PCR. RNA quantity was measured using the Quant-iT RiboGreen RNA Assay Kit (Thermo Fisher Scientific), and RNA quality was assessed on the Fragment Analyzer automated CE system (Agilent Technologies) with the 471 RNA Standard Kit. Ribosomal Integrity Number (RIN) values were recorded for each sample.

### Illumina transcriptome library preparation and sequencing

Except for one sample with 65 ng RNA input, 150 ng of quantified total RNA was used for first-strand cDNA synthesis with the SuperScript IV VILO Mastermix and ezDNase enzyme (Invitrogen). Sequencing libraries were prepared using the NEBNext Ultra II FS DNA Library Prep Kit for Illumina (New England BioLabs), following the manufacturer’s protocol. Briefly, cDNA was enzymatically fragmented for 15 minutes at 37°C, followed by adapter ligation using NEBNext Ultra II Multiplex Oligos for Illumina and purification with MagSi-NGSPrep Plus beads (Magtivio BV). Adapter-ligated fragments were enriched by 10 cycles of PCR using NEBNext Multiplex Oligos, and PCR products were purified twice with MagSi-NGSPrep Plus beads. Library quality, including fragment size and adapter dimer removal, was confirmed using a Fragment Analyzer system (Agilent Technologies) with the 473 Standard Sensitivity Kit. Sequencing aimed for a read depth of up to 400 million paired-end reads per sample. Libraries were sequenced on an Illumina NextSeq 550 using the NextSeq High Output Kit v2.5 (2 × 150 bp, paired-end).

### Sequencing reads library processing

The RNA sequencing generated a total of 1.11 × 10^9^ reads. Trimming of raw reads was performed using fastp v0.23.4 [44], followed by quality assessment with multiqc v1.23 [45]. Reads were pseudo-aligned to the predicted coding sequences of the respective parental strain using kallisto v0.50.1 [46], with 99 bootstrap samples. K-mer sizes ranging from 17 to 31 were evaluated, and a final k-mer size of 31 was selected based on the highest average percentage of pseudo-aligned and uniquely mapped reads in both samples and negative controls. A median of 5.5 × 10^6^ reads per sample were pseudo-aligned (61.75%, excluding negative controls). Downstream analyses were conducted in R v4.5.1 [47] with Rstudio v2025.09.1 [48]. Count estimates and bootstrap replicates were imported using tximport v1.36.0 [49] and summarized at the gene level. Reads mapping to ribosomal RNA, transfer RNA, and elongation/initiation factors were excluded. Samples (*n* = 12) and genes with low coverage (*n* = 1016) were filtered out. The final dataset included 30 samples (*n* = 3–5 per group; 14 *flhC*+ and 16 *flhC*−), 9.67 × 10^5^ reads (median of 2.12 × 10^4^ reads per sample), and 1605 genes. The count matrix sparsity was controlled at 0.112. To normalize for sequencing depth disparities and compositional biases [50], a centred log-ratio transformation was applied. As the logarithm is undefined for zero counts, a pseudo-count was added to zero values. This pseudo-count was calculated per sample as the product of the constant e^−1^ and the minimal detection limit. The mean and variance of log-ratios across all bootstrap replicates were used to quantify gene relative expression and technical variability, respectively.

### Transcriptome analysis

Singular value decomposition was applied to embed samples into a low-dimensional space. Permutational Multivariate Analysis of Variance (PERMANOVA) was conducted using the vegan package v2.7.1 [51] to evaluate the contribution of covariates to sample clustering within this space. A gene-association matrix was separately computed for the *flhC+* and *flhC−* groups using the propr package v5.1.17 [52]. This analysis included an equal number of samples per group (*n* = 14) and retained only genes with counts equal to or exceeding the median per-sample read count in at least 50% of samples. The proportionality coefficient ρ was calculated among gene-pairs, and false discovery rates (FDR) were estimated using 999 permutations. Robust associations were retained by selecting only pairs with ρ above 0.75. Resulting gene association networks were analysed using tidygraph v1.3.1 [53] and ggraph v2.2.2 [54]. Network properties including size, order, modularity, efficiency, node degree centrality, and clustering by greedy optimization were computed. Enrichment of plant-growth-promotion (PGP) terms within the *flhDC* cluster was tested using clusterProfiler v4.16.0 [55]. To assess the effects of *flhC* status, media type, and their interaction on gene expression, a linear mixed-effects model was fitted for each gene using the nlme package v3.1–168, with the formula: ~ *flhC* * media. Strain identity was included as a random intercept to control for residual variation between strains. The model incorporated combined variance functions for technical variability from pseudo-alignment and heteroscedastic residuals. Local FDR were controlled at α = 0.05 using the qvalue package v2.40.0 [56]. Pairwise post hoc comparisons of *flhD* and *flhC* expression levels were performed via the comparisons function in marginaleffects v0.30.0 [57]. Pathway analysis was conducted using generalized least squares implemented in nlme at the fifth hierarchical level of the PLaBAse ontology, based on gene-wise estimates from the linear mixed-effects models. Each gene’s squared estimated standard deviation was included as a fixed variance parameter to model heteroscedasticity.

### FlhDC-box analysis

Upstream regions (200 bp) of all predicted coding sequences in the LR140 genome were extracted using BEDTools v2.31.1 [58]. A motif corresponding to the FlhDC binding site (FlhDC-box) was identified from class II flagellar genes with MEME v5.5.7 [59], using the *E. coli* consensus sequence ANNAN18TN(A/T)TN6(A/T)T [60] as the initial seed. The resulting motif was then used to screen all upstream regions with FIMO v5.5.7 [61]. High, medium, and low confidence matches *q*-value thresholds were calculated to control the rate of false discoveries at 1, 10, and 20 matches over 4616 sequences.

### *In planta* bioassays

*L. japonicus* seeds were manually scarified using sandpaper, surface sterilized with 11% sodium hypochlorite (NaClO) for 15 minutes, washed five times in sterile distilled water, and incubated in sterile distilled water for 2 hours. Imbibed seeds were germinated on B5 medium (3.3 g/L Gamborg B5 salts, 20 g/L sucrose, 10 g/L Bacto agar) under dark conditions at 24°C for 3 days, followed by 3 days under light at 24°C. Seedlings were then transferred to Duchefa tissue culture boxes (Duchefa Biochemie, Netherlands), each containing 300 g of autoclave-sterilized quartz sand and supplemented with 30 ml of half-strength Hoagland’s solution. Six plants were grown per box. For AMF treatments, *R. irregularis* DAOM197198 spores (Type C, Agronutrition, Toulouse, France) were applied at a density of 500 spores per plant. Each box was sealed with a hermetic but gas-permeable lid to preserve sterility while allowing gas exchange. *Acidovorax* strains were cultured in 50% strength TSB at 21°C overnight until reaching exponential phase and diluted to an OD^600^ of 0.01. A total of 25 ml of bacterial suspension was applied per box. For each bacterial strain, six replicate boxes were prepared. Plants were cultivated under a long-day photoperiod (16 h light / 8 h dark) at 23°C/21°C (day/night temperature). After 5 weeks, plant roots were harvested to quantify bacterial and fungal colonization.

### Quantification of root colonization by *Acidovorax* bacteria

Roots were harvested and weighed for enumeration of bacterial colonization, and approximately 20 mg of tissue per sample was homogenized in 1 ml of sterile distilled water. Serial dilutions of the homogenate were prepared in sterile water, and 100 μl aliquots from each dilution were plated on 50% TSB agar. Colony-forming units (CFUs) were enumerated after 3 days of incubation. Statistical analysis of colonization levels was conducted using binomial mixed-effects models implemented in the lme4 package v1.1–37, with post hoc pairwise comparisons of marginal means performed using the marginaleffects package v0.24.0. FDR were controlled using the Benjamini–Hochberg procedure.

## Results

### Closely related strains differ in *flhC* and swimming motility

Our selection of *A. delafieldii* strains LR140 (*flhC*+) and LR124 (*flhC*−) was informed by a previous study [21], which reported divergent effects on *Lotus japonicus* growth upon re-inoculation: LR140 (*flhC*+) had a negative effect, whereas LR124 (*flhC*−) promoted host growth. Despite nearly identical genome sizes (5.02 Mb) and a high ANI (99.968%), the strains differ slightly in gene content. Specifically, LR124 lacks *flhC* and two additional putative coding sequences, while LR140 lacks a *lysR* homolog and one predicted gene containing a Pfam domain of unknown function (Table S3). Genomic context suggests that the *lysR* gene in LR124 may have arisen from a duplication event.

**Figure 1:**
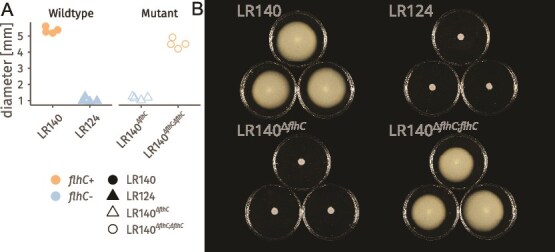
*flhC*- strains are non-motile A) halo diameter in motility assay in 3% soft agar media, showing the loss of motility due to lack of *flhC* (strains LR124 and LR140*^ΔflhC;flhC^*) n = 4. B) Images of three representative petri dishes per strain exemplifying the halo diameter measured in a).

Given that *flhC* encodes a key regulator of flagellar assembly genes, and that flagella contribute to diverse behaviours in planta, we aimed to use these strains to dissect how this regulatory checkpoint modulates bacterial perception of and response to plant- and mycorrhiza-derived metabolites. To isolate the effects of *flhC*, independent of the other genomic differences between LR140 and LR124, we generated an in-frame *flhC* deletion mutant of LR140 (LR140*^ΔflhC^*) and a corresponding reconstituted strain (LR140*^ΔflhC;flhC^*). Motility assays on soft agar confirmed that LR124 and LR140*^ΔflhC^ (flhC−)* were non-motile, while LR140 and LR140*^ΔflhC;flhC^* (*flhC*+) retained motility (Fig. 1A and B).

### *flhC* shapes strain-specific and mycorrhiza-modulated transcriptomes

**Figure 2:**
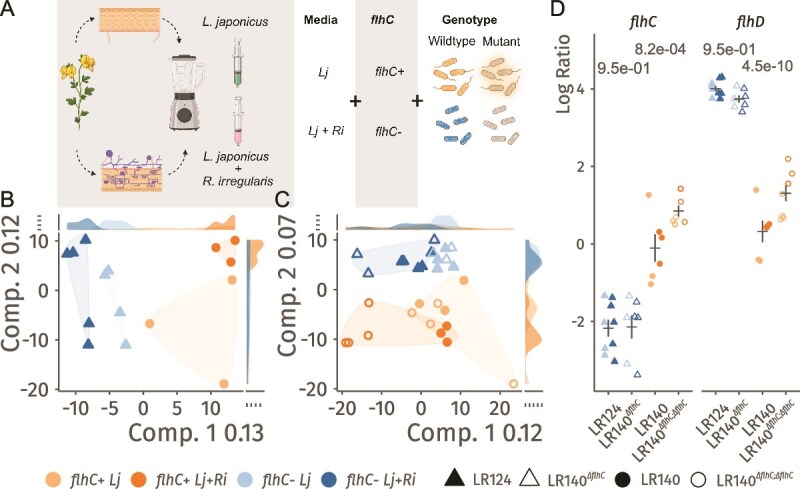
*flhC* and *L. japonicus* root metabolomes create distinct transcriptional profiles. A) Depiction of the experimental design. Non-colonized and *R. Irregularis*-colonized *L. japonicus* roots were homogenized and sterilized by progressive filtering to 0.22 μm). LR140, LR124, LR140*^ΔflhC^* and LR140*^ΔflhC;flhC^* were cultivated in minimal media based on M9 supplemented with these root extracts (*Lj* or *Lj + Ri*) for 24 h prior to cell harvesting and RNA extraction. For each combination of strain and growth media, RNA from five biological replicates was sequenced. Created in BioRender. Siani, R. (2026) https://BioRender.com/c67h242 B) dimensionality reduction of the transcriptomes shows that wildtype strains (LR140 and LR124) group by the presence or absence of *flhC* (first component) and by the growth media (*Lj* or *Lj + Ri*; first and second component). First and second components are projected on the x and y axis. C) Dimensionality reduction including wildtype and mutant strains (LR140*^ΔflhC^* and LR140*^ΔflhC;flhC^*). And showing separation due to presence or absence of *flhC* (second component) and media type (*Lj* or *Lj + Ri,* first component). D) Expression levels (log ratio) of the genes *flhC* and *flhD* in the four strains across the two media conditions. Significance of the differences between media type for *flhC*+ and *flhC*- strains for each gene was tested using linear mixed models, followed by a post-hoc comparison of marginal effects. For transcriptomics, three to five biological replicates were used per combination of strain and media.

To investigate how the absence of *flhC* affects the bacterial transcriptional response to plant-derived metabolites, we profiled the transcriptomes of LR124, LR140, LR140*^ΔflhC^*, and LR140*^ΔflhC;flhC^* after cultivation in mechanically shaken, minimal medium supplemented with root extracts from either non-mycorrhizal (*Lj*) or *R. irregularis*-colonized (*Lj + Ri*) *L. japonicus* roots (Fig. 2A). We did not observe significant differences in growth rates among strains and between media. Dimensionality reduction of the transcriptomic data revealed that LR124 and LR140 samples were separated along the first dimension, which accounted for 13% of the total variance (PERMANOVA R^2^ = 0.125) (Fig. 2B). A weaker, yet statistically significant separation was also observed based on media type (R^2^ = 0.094), indicating that both strain identity and root metabolite composition influenced the transcriptional profiles.

Including the *flhC* deletion and complemented strains in the analysis revealed four distinct transcriptomic clusters (Fig. 2C). In this broader dataset, separation according to media type explained 12% of the variance (R^2^ = 0.076), while *flhC* status accounted for 7% (R^2^ = 0.069), with particularly clear clustering based on the presence or absence of *flhC*. Notably, both LR124 and LR140*^ΔflhC^* (*flhC*−) exhibited elevated expression of *flhD* compared to the *flhC*+ strains (LR140 and LR140*^ΔflhC;flhC^*, Fig. 2D), suggesting a compensatory regulatory mechanism in response to the loss of *flhC*.

In *flhC*− strains, *flhD* expression remained stable across media types. In contrast, in *flhC*+ strains, *flhDC* expression was approximately 2-fold higher (*P* < 0.05) when cultured in *Lj + Ri* media, raising the possibility that root colonization by *R. irregularis* may influence bacterial motility through the *flhDC* regulatory pathway.

### *flhC* loss reduces network connectivity and efficiency

**Figure 3:**
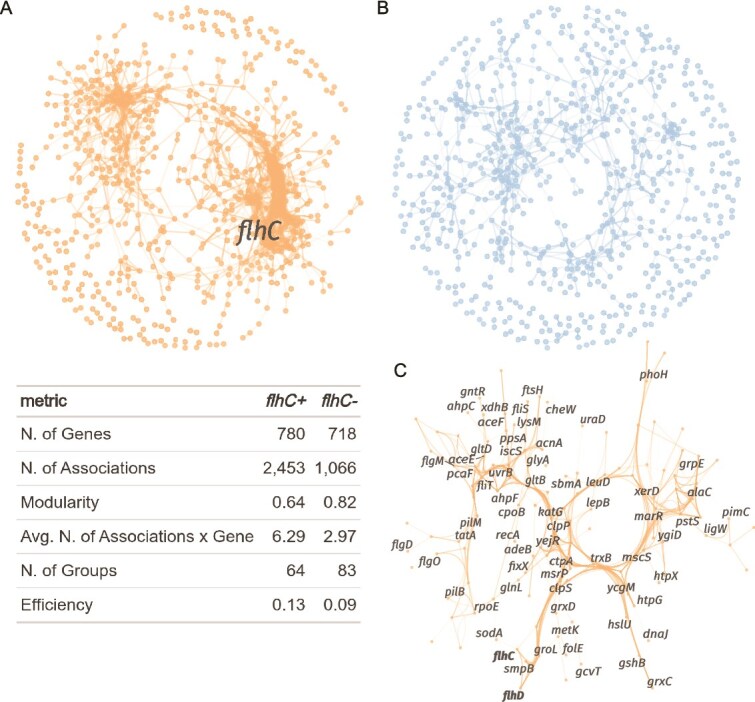
Genes expressed in *flhC*- strains show a smaller, more modular network, with fewer associations per genes. The gene-association networks are based on the proportionality coefficient ρ. Graph properties are listed in the table. A) *flhC*+ network is shown in orange, B) *flhC*- in blue. Links represent associations between genes (only ρ > 0.75 are included). C) Sub-network showing the cluster of genes more closely associated with *flhC*. For network inference, 14 *flhC*+ (LR140 and LR140*^ΔflhC;flhC^*) and 14 *flhC*- (LR124 and LR140*^ΔflhC^*) biological replicates were separately analysed.

We compared gene-association networks of *flhC*+ (LR140 and LR140*^ΔflhC;flhC^*) and *flhC*− (LR124 and LR140*^ΔflhC^*) strains to evaluate how *flhC* influences transcriptional coordination. Using proportionality-based associations (*ρ* ≥ 0.75) among consistently expressed genes (*n* = 1065 for *flhC*+, n = 1068 for *flhC*−), we reconstructed and analysed gene co-expression networks. This approach recovered strong associations among a large proportion of genes, with 780 nodes in the *flhC*+ network (Fig. 3A) and 718 in the *flhC*− network (Fig. 3B). However, genes in the *flhC*- network exhibited, on average, less than half the number of connections (i.e. lower degree centrality) compared to the *flhC*+ network, resulting in sparser connectivity overall.

In addition to reduced connectivity, the *flhC*− network exhibited increased modularity, with a greater number of smaller and more distinct clusters, and lower global efficiency, reflecting a reduced capacity for information exchange across the network. These findings suggest that transcriptional coordination is weakened in the absence of *flhC*, and regulatory modules become more fragmented. In bacteria, the modular organization of gene networks enhances their capacity to withstand and adapt to environmental fluctuations and novel conditions. In contrast, *flhC* supports tighter co-regulation among functionally related genes, enhancing network integration and efficiency.

### The *flhC*-associated gene cluster links motility to stress tolerance and plant-interaction traits

To assess whether differences in gene co-expression may impact plant interactions, we used PLABase [34], a curated ontology of bacterial genes involved in plant association and plant growth promotion (PGP). PLABase organizes genes into seven hierarchical levels (PGP0–PGP6), encompassing direct effects on the host and indirect traits influencing microbial fitness within the plant microbiome. For consistency, we refer to all traits in the ontology as PGP-related.

In the *flhC*+ (LR140 and LR140*^ΔflhC;flhC^*) network, *flhDC* was embedded within a cluster of 127 genes (Fig. 3C), enriched for PGP terms related to motility and chemotaxis (14 genes related to flagellar and pili assembly, and control), universal stress response (12 genes) and neutralization of abiotic stress (33 genes). Other genes found in the cluster were involved in the organic acid, lipid, and amino acid metabolism, and biofilm regulation.

Notably, *flhC* was strongly co-expressed with six genes: *clsP* (Clp protease adaptor), *groES* (co-chaperonin), *groL* (chaperonin), *smpB* (SsrA-binding protein), *sodA* (superoxide dismutase), and a dioxygenase superfamily protein. Together with *flhD*, the co-expression network included *dnaK* (molecular chaperone), and genes coding for 3-hydroxyacyl-CoA dehydrogenase, the endopeptidase La.

These results suggest that *flhC* integrates motility regulation with broader transcriptional programs, likely relevant for bacterial fitness in the plant-associated niche, linked to stress resilience, redox balance, and metabolite processing.

### FlhDC directly interact with a small fraction of genes

To distinguish between direct and indirect effects of FlhDC on gene transcription, we identified a motif matching (*P* = 6.21e-04) *E. coli* consensus binding site ANNAN_18_TN(A/T)TN_6_(A/T)T in the upstream region of known FlhDC regulatory targets (*flgA, flgB, flhB, fliA, fliE, fliF, fliL, e* = 1.9e-005, Supp. Fig. S1A).

We screened the upstream regions of all predicted coding sequences in *A. delafieldii* LR140 and detected potential binding sites of FlhDC (FlhDC-box, Suppl. Table S4). High confidence matches (10, *q* < 0.05) included the previously mentioned known regulatory targets and two more genes, coding for a DNA-binding response regulator of the OmpR family and a hypothetical protein. Medium confidence matches (7, *q* between 0.05 and 0.1) included a transcriptional regulator of the LysR family. Lower confidence matches (12, *q* between 0.1 and 0.15) included the flagellar motor stator protein MotA, and transcriptional regulators of the GntR and MarR family. In the gene-association network, we did not see significant association (*rho* > 0.75) between expression levels of these genes and *flhDC*, suggesting a more complex and conditional regulation. However, looser association thresholds (ρ ~ 0.5) suggest a potential interaction between *flhDC* and *marR, ompR*, and genes coding for a lipoprotein and a tetratricopeptide repeat-containing protein.

### Absence of *flhC* is associated with nutrient mobilization

To elucidate the regulatory role of *flhC* putatively downstream of potential primary targets or of secondary effects of its loss, we compared gene and pathway expression between *flhC*+ strains (LR140 and LR140*^ΔflhC;flhC^*) and *flhC*− strains (LR124 and LR140*^ΔflhC^*), using PLABase to annotate potential functions related to plant-microbe interactions.

**Figure 4:**
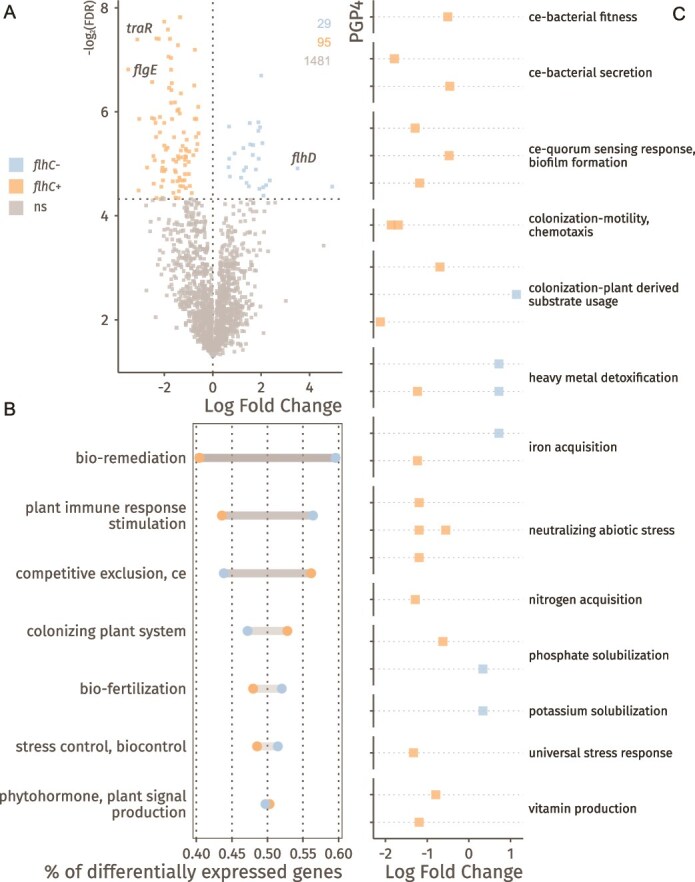
Stress response, substrate preferences, biofilm formation and secretion are modulated by *flhC*. A) Differential gene expression (log-fold changes; x axis), between *flhC*+ (LR140, LR140*^ΔflhC^*) and *flhC*- (LR124, LR140*^ΔflhC;flhC^*) strains, controlling for media type and between strain variances. Significance threshold was set to control local false discovery rates below 0.05 and is reported on the y axis (negative logarithm of the local FDR). Coloured points represent genes whose expression significantly differs between conditions. B) Differentially expressed genes (*flhC+* vs *flhC−*) assigned to the broad PGP2 level of the PLABase ontology, showing general trends in the expression of PGP traits. For each category, the number of differentially expressed genes is normalized to the total number of genes assigned to that category. C) Pathway analysis showing the estimated coefficients (x axis) for each significantly (local FDR controlled at 0.05) differentially expressed PGPs pathway. Each point represents a PGPs pathway and is assigned to a broader category of the ontology, shown on the y axis of the plot.

Overall, 124 of 1605 genes (~7%) were differentially expressed between *flhC*- and *flhC*+ strains across both media conditions (q < 0.05; Fig. 4A, Supplementary Data S3). Compared to *flhC*+ strains, *flhC*- strains showed reduced expression of genes associated with competitive exclusion and colonization, and increased expression of genes involved in nutrient mobilization and environmental remediation (Fig. 4B, Supplementary Data S4).

Among the 124 differentially expressed genes, 29 had significantly higher expression levels in *flhC*- strains. These genes were linked to plant-derived substrate utilization, resistance to abiotic stress, xenobiotic degradation, and solubilization of phosphate and potassium including *cysD* (sulfur assimilation), *ftsA* (cell division), *gspE* (type II secretion), *hisD* (histidine biosynthesis), *ilvE* (brached-chain amino acids metabolism), *nadE* (NAD+ biosynthesis), *nuoF* (NADH-quinone oxidoreductase), *ugpA* (glycerol-3-phosphate ABC transporter), and genes encoding a putative permease, a GGDEF domain-containing protein, and an acyltransferase family protein.

The remaining 95 differentially expressed genes were more highly expressed in *flhC*+ strains. Beyond the expected enrichment in flagellar assembly and chemotaxis genes, this group included genes involved in substrate metabolism, abiotic stress resistance, biofilm formation, quorum sensing, secretion, surface attachment, and cell envelope remodelling.

To assess the broader impact of *flhC* on plant-microbiome interaction phenotypes, we analysed pathway-level effects on PGP-related traits (PLABase level 5; Fig. 4C, Supplementary Data S5). The *flhC*- condition was associated with increased expression of pathways related to phosphate and potassium solubilization via succinate biosynthesis, iron acquisition, and glycerol-3-phosphate transport. Several pathways were instead less expressed, including general secretion systems, c-di-GMP signalling, autoinducer perception, alanine and fumarate metabolism, hemophore production, vitamin B6 biosynthesis, salinity resistance, nitrogen regulation, phosphate solubilization, and the carbohydrate starvation response.

These findings suggest that *flhC* acts as a regulatory hub, balancing motility and colonization with metabolic flexibility and nutrient mobilization.

### Arbuscular mycorrhiza-induced root metabolite composition reprograms *Acidovorax* substrate utilization and interaction traits

**Figure 5:**
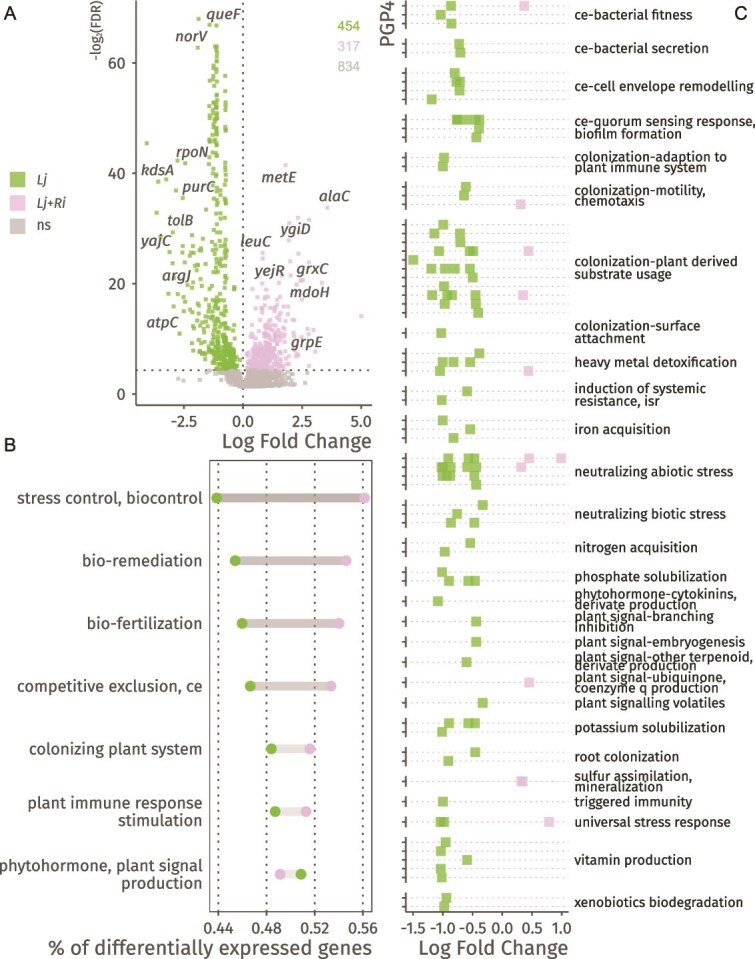
*Lj + Ri* mediates gene expression related to plant-derived substrate usage, stress response, type II secretion systems and chemotaxis. A) Differentially expressed genes (log-fold changes; x axis) observed in all strains (LR140, LR124, LR140*^ΔflhC^* and LR140*^ΔflhC;flhC^*) due to cultivation in *Lj* and *Lj + Ri* medium. Significance threshold was set to control local false discovery rates below 0.05 and is reported on the y axis as the negative logarithm of the local FDR. Coloured points represent genes whose expression significantly differs between conditions. B) Differentially expressed genes (*Lj* vs *Lj + Ri*) assigned to the broad PGP2 level of the PLABase ontology, showing general trends in the expression of PGPs. For each category, the number of differentially expressed genes is normalized to the total number of genes assigned to that category. C) Pathway analysis showing the estimated coefficients (x axis) for each significantly (local FDR controlled at 0.05) differentially expressed PGPs pathway. Each point represents a PGPs pathway and is assigned to a broader category of the ontology, shown on the y axis of the plot.

We further investigated how *A. delafieldii* strains LR140, LR124, LR140*^ΔflhC^*, and LR140*^ΔflhC;flhC^* respond transcriptionally to media enriched with extracts from *L. japonicus* roots colonized by *R. irregularis* (*Lj + Ri*) compared to extracts from non-colonized roots (*Lj*). Across strains, approximately 48% of expressed genes (771 of 1601) showed significant differential expression in the *Lj + Ri* condition: 454 genes were downregulated and 317 upregulated (Fig. 5A, Supplementary Data S3).

Upregulated genes were enriched in PGP categories (Fig. 5B, Supplementary Data S4), such as stress control (abiotic stress resistance), colonization (motility, chemotaxis, plant-derived substrate usage), competitive exclusion (quorum sensing and biofilm formation), and fertilization (phosphate and potassium solubilization). At higher resolution (Fig. 5C, Supplementary Data S5), this response included increased expression of genes related to ubiquinone/coenzyme Q biosynthesis, sulphate and thiosulfate transport, thioredoxin and protease production, chemotaxis two-component signalling, dimethyl sulphide degradation, malate and glycine metabolism, and resistance to copper and beta-lactam antibiotics. Conversely, *Lj + Ri* treatment caused downregulation of genes associated with plant-derived substrate metabolism and abiotic stress responses. Additionally, pathways related to general secretion, biofilm formation, and autoinducer perception were suppressed.

### Loss of *flhC* alters the bacterial transcriptional response to arbuscular mycorrhiza-modified root metabolites

**Figure 6:**
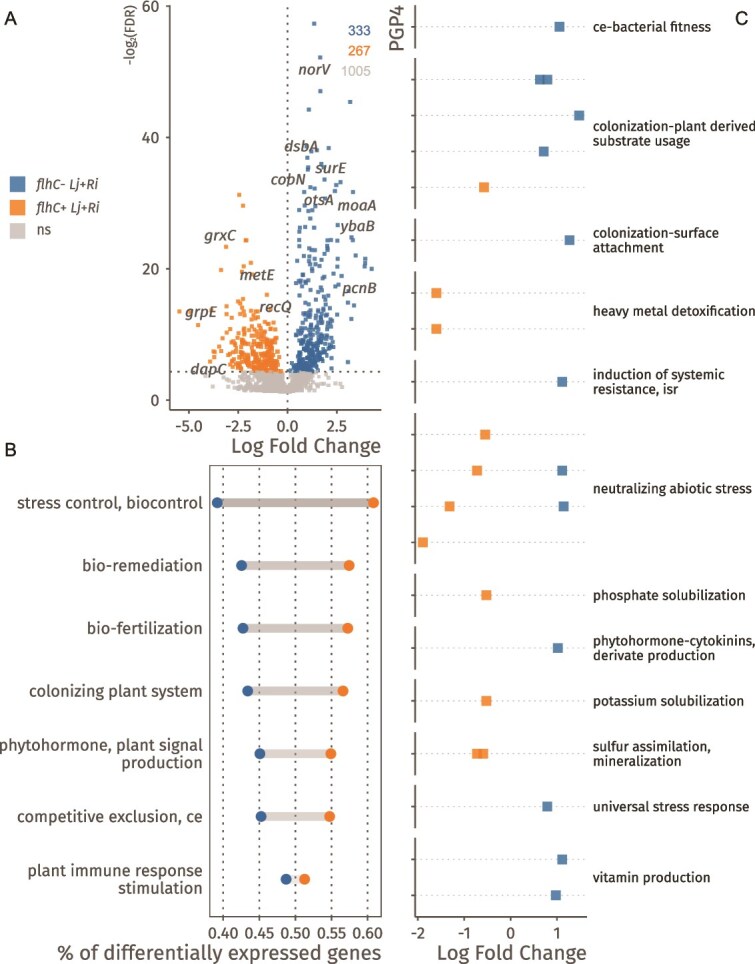
*flhC* modulates the response to *Lj + Ri*, regulating genes related to surface attachment and nutrient utilization. A) Differentially regulated genes between *flhC+* (LR140, LR140*^ΔflhC^*) and *flhC-* (LR124, LR140*^ΔflhC;flhC^*) in response to the *Lj + Ri* medium, controlling for between strain variances. Significance threshold was set to control local false discovery rates below 0.05 and is reported on the y axis as the negative logarithm of the local FDR. Coloured points represent genes whose expression significantly differs between conditions. B) Differentially expressed genes (*flhC+* vs *flhC-* in *Lj* + *Ri* media) assigned to the broad PGP2 level of the PLABase ontology, showing general trends in the expression of PGPs. For each category, the number of differentially expressed genes is normalized to the total number of genes assigned to that category. C) Pathway analysis showing the estimated coefficients (x axis) for each significantly (local FDR controlled at 0.05) differentially expressed PGPs pathway. Each point represents a PGPs pathway and is assigned to a broader category of the ontology, shown on the y axis of the plot.

We assessed whether the absence of *flhC* alters the transcriptional response of *A. delafieldii* to root extracts from *R. irregularis*-colonized *L. japonicus* (*Lj + Ri*). Comparing *flhC*- strains (LR124 and LR140*^ΔflhC^*) with *flhC*+ strains (LR140 and LR140*^ΔflhC;flhC^*), we found significant differential regulation in 37% of genes (600 of 1605), including 333 upregulated and 267 downregulated transcripts (Fig. 6A, Supplementary Data S3).

The most pronounced differences affected genes involved in stress tolerance and colonization traits (Fig. 6B, Supplementary Data S4). Downregulated pathways in *flhC*– strains included dimethyl sulphide degradation, succinic acid synthesis, glycine–betaine metabolism, as well as salinity-, oxidative-, and thermal stress responses, and glycine degradation (Fig. 6C, Supplementary Data S5).

In contrast, upregulated genes in *flhC*− strains included those associated with stress responses and utilizing specific plant-derived compounds (formate, C4-dicarboxylates, pyrimidines, fumarate). Additionally, *flhC*− strains showed enhanced biosynthesis of vitamins and cofactors (xanthine, cobalamin, niacin, riboflavin), increased expression of surface attachment proteins, and induction of aerobic respiration operons: *nuo* (type I NADH:quinone oxidoreductase), *ndh* (type II NADH dehydrogenase), and cytochrome c oxidase (*cco*).

### *flhC* reduces root colonization by *Acidovorax*

**Figure 7:**
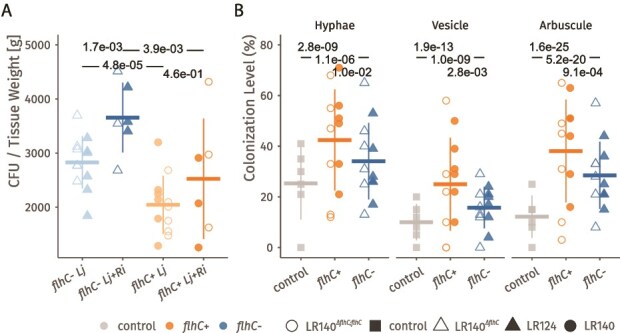
*flhC* is detrimental for colonization of *L. japonicus* roots. (A) Number of recovered colony forming units (CFU) per gram of root tissue, showing the differences between *flhC+* (LR140, LR140*^ΔflhC^*) and *flhC-* (LR124, LR140*^ΔflhC;flhC^*). The horizontal and vertical segments represent mean and standard deviation. Linear mixed models were used to test the significance of differences, followed by a post-hoc comparison of marginal means and Benjamini-Hochberg correction. B) Levels of root colonization by *R. irregularis* hyphae, arbuscules and vesicles (number of intersections counted normalized to 100). The horizontal and vertical segments represent mean and standard deviation. Binomial mixed models were used to test the significance of differences, followed by a post-hoc comparison of marginal means and Benjamini-Hochberg correction. Twelve biological replicates (six with and six without *R. Irregularis* colonization) were used for each strain.

To assess the role of *flhC* in root colonization, *L. japonicus* plants were inoculated with *flhC*+ (LR140, LR140*^ΔflhC;flhC^*) and *flhC*- (LR124, LR140*^ΔflhC^*) strains, and bacterial colonization was measured after five weeks by colony count per tissue weight after streaking serial dilutions of root homogenate onto agar dishes. Strains lacking *flhC* showed significantly higher colony counts, indicating higher root colonization than *flhC*+ strains (Fig. 7A). Furthermore, the *flhC*- strains exhibited increased colonization when co-inoculated with *R. irregularis*, indicating a positive interaction, potentially because fungal hyphae may facilitate movement of non-flagellated bacteria.

*R. irregularis* colonized (hyphae, vesicles, arbuscules) the roots more extensively when co-inoculated with *A. delafieldii* (Fig. 7B). This effect was stronger with *flhC*+ than *flhC*-strains.

## Discussion

Our findings suggest that *flhC* coordinates multiple physiological functions beyond flagellar assembly, acting as a key regulatory node linking transcriptional programs related to motility, metabolism, and environmental adaptation in *A. delafieldii*. The loss of *flhC* broadly reprogrammed transcription, affecting stress response, nutrient utilization, and pathways involved in cell envelope construction.

Consistent with other species and with the conserved role of the FlhDC complex as master regulator of motility across diverse Pesudomonadota [62–64], we confirmed that deletion of *flhC* results in lack of swimming motility in *A. delafieldii flhC-* strains (LR124, LR140*^ΔflhC^*). Transcriptome analyses were performed using a minimal medium supplemented with extracts from mycorrhizal (*Lj + Ri*) and non-mycorrhizal (*Lj*) *L. japonicus* roots. This approach revealed clear *flhC*-dependent transcriptional profiles and responses to the *Lj + Ri* medium across wildtype and mutant strains. Minor differences within strain pairs were attributed to a twofold higher expression of *flhDC* in the complemented mutant (LR140*^ΔflhC;flhC^*) compared to LR140, as well as elevated *flhD* expression in *flhC*- strains relative to *flhC*+ strains (LR140, LR140*^ΔflhC;flhC^*).

Regulation of *flhDC* is known to integrate multiple environmental and cellular signals including temperature, osmolarity, nutritional status, and quorum sensing [65–68]. In LR140 and LR140*^ΔflhC;flhC^* the *flhDC* expression level was higher in *Lj + Ri* medium than in *Lj* medium, possibly because of relatively higher expression levels of the H-NS encoding gene, and *ompR*, which are known positive regulators of *flhDC* [69–71]. Negative feedback loops coordinate FlhDC activity and flagellar assembly [72]. We propose that these feedback mechanisms remain inactive in the absence of *flhC* and therefore the FhlDC complex and its downstream products, resulting in increased *flhD* expression and intracellular accumulation of free FlhD protein. Our study cannot fully disentangle effects caused directly by *flhC* deletion from those possibly resulting from elevated *flhD* expression. Although FlhD was previously suggested to have independent regulatory function [13], more recent evidence questions this role [73, 74] and current consensus supports that *flhC* deletion alone is sufficient to disrupt FlhDC complex regulatory activity [74].

The transcriptional changes we observed were accompanied by major shifts in gene-association networks. In the *flhC*− strains, these networks were less connected, with reduced information flow and more distinct modules, showing that removing this single regulator causes broad reorganization of the network. Modularity is key to regulatory network adaptability [75, 76], and a relaxation of regulatory control may enhance the capacity of bacteria to colonize novel environments.

Our results further show that *flhDC* is co-expressed with genes enriched for functions related to stress tolerance, motility and chemotaxis, organic acid metabolism, and biofilm regulation. This cluster included several genes known to be associated with *flhDC* in other bacterial species. The heat shock proteins DnaK, DnaJ, and GrpE, as well as the histone-like H-NS have long been known to participate in the regulation of flagellar assembly in response to temperature [77] and acidity [78]. Interestingly, *pilM, pilB* and the pilin encoding gene were also found in this cluster, in line with previous observations of flagella-pili co-regulation in the closely related *A. citrulli* [79].

The relatively large cluster size compared to the number of differentially expressed genes suggests many transcriptional changes to be indirect, potentially mediated through other regulators, and also reflecting resource reallocation from flagellar assembly [80]. In fact, our examination of the putative binding sites for FlhDC points to a limited number of directly regulated genes, which is consistent with the findings for *E. coli* [74]. The 34 base pairs motif we identified includes the canonically conserved base pairs A^1^, A^4^, T^23^, W^25^, T^26^, W^33^, and T^34^ [60]. Notably, we detected a sequence resembling this motif in the promoter of a OmpR-like encoding gene. Regulation of FlhDC by the osmolarity stress response regulator OmpR was first detected in *E. coli*, and then confirmed in a few other genera [70, 81]. Our findings suggest that, in *A. delafieldii* LR140, a feedback loop may allow FlhDC to regulate OmpR.

We next examined how transcriptional changes linked to *flhC* absence could modulate bacterial interactions with the plant host. Differential expression was observed across numerous PGP pathways. Notably, *flhC* deletion correlated with repression of nitrogen cycle regulatory elements, consistent with findings in the plant pathogen *A. citrulli* [82]. Downregulation of cyclic di-GMP (c-di-GMP) signalling pathways is particularly relevant, as modulation of this global regulator is implicated in transitions from free-living to host-associated lifestyles [83]. Concurrent upregulation of iron acquisition, potassium and phosphate solubilization, surface adhesion, and vitamin biosynthesis pathways may also facilitate this lifestyle shift. Surface adhesion and nutrient acquisition mechanisms contribute to competitive exclusion [84, 85], while nutrient solubilization and vitamin synthesis can benefit both plant hosts and AMF [86, 87]. Increased synthesis of surface adhesion proteins may also partially compensate for the loss of flagella, which participate in attachment in various bacterial species [88].

*A. delafieldii*’s alteration of transcriptional response to the presence of *R. irregularis* align with substantial shifts in the secondary metabolome relative to non-mycorrhizal roots, including an enrichment of bioactive polyphenols [30]. However, a direct link to specific metabolites is currently hampered as most metabolites affected by the symbiosis remained structurally uncharacterized because of a lack of corresponding compounds in metabolite databases and the generally high proportion of recorded but unidentified metabolites in plants [30]. In all tested *A. delafieldii* strains, we observed differential expression of genes involved in substrate utilization and assimilation of nutrients and micronutrients, potentially reflecting underlying chemical differences in the root extracts.

Among the transcriptional responses, type II secretion systems (T2SS), frequently employed by plant-associated bacteria to export hydrolytic enzymes [89], were downregulated in the *Lj + Ri* medium. This is notable considering that recent findings have shown repression of T2SS can mitigate opportunistic infections and preserve microbiome stability in leaf tissues [90, 91]. Future work could determine whether the observed reduction in T2SS expression is driven directly by fungal metabolites or indirectly via AMF-induced reprogramming of the host plant’s metabolic status.

Additionally, we demonstrated that *flhC* modulates the bacterial response to the *Lj + Ri* metabolite mix. Specifically, *flhC-* strains exhibited upregulation of the *nuo, ndh*, and *cco* operons, central to aerobic respiration. This is consistent with findings in *E. coli*, where FlhDC represses aerobic respiration and promotes anaerobic respiratory pathways [13]. The absence of FlhC, and thereby disruption of the FlhDC complex, appears to relieve this repression in *A. delafieldii*, leading to a shift toward aerobic metabolism in response to the *R. irregularis* symbiosis-altered root metabolism.

Finally, we evaluated the root colonization capacity of each *A. delafieldii* strain and its potential influence on *R. irregularis* colonization. Our data show that *flhC*- strains consistently achieved higher root colonization levels compared to *flhC*+ strains, with the lowest recovery observed for LR140*^ΔflhC;flhC^*, which also exhibited the highest *flhDC* expression. This supports our initial hypothesis that flagellar expression may pose a disadvantage during plant association, potentially due to the substantial energetic burden of flagellum biosynthesis and maintenance, as well as the immunogenicity of flagellin, which can trigger host defence responses. Transcriptomic data further corroborate these observations, revealing that *flhC*- strains upregulated pathways involved in iron and mineral acquisition and genes encoding surface attachment factors, traits likely to enhance competitive colonization within the rhizosphere and root tissues.

Co-inoculation with *Acidovorax* significantly enhanced *R. irregularis* colonization, with an even stronger effect observed for arbuscule formation. Co-inoculation with *flhC+* strains was especially beneficial. Given the lower colonization densities of these strains, this trend may reflect strain-specific behaviours or signalling within the plant or directly with the fungus, rather than overall bacterial load. The role of bacterial–fungal interactions in shaping symbiosis outcomes is supported by studies showing that commensal bacteria can modulate root colonization by AMF and contribute to host fitness via interkingdom crosstalk [92–94]. Notably, only *flhC*- strains exhibited a significant increase in root colonization when co-inoculated with *R. irregularis*, indicating a possible synergistic effect of AMF-derived cues on bacterial fitness for example by promotion of bacterial movement towards the plant along hyphae [95]. This is consistent with reports that AMF symbiosis alters the composition and functional potential of the root-associated microbiome in *L. japonicus* [96], potentially through shared or complementary suppression of host immunity [97].

In this context, the downregulation of the *Acidovorax* T2SS in *Lj + Ri* medium may be of functional relevance. T2SS-secreted effectors in plant-associated bacteria modulate host perception of MAMPs, such as flg22 [98]. Downregulation of T2SS may reduce the capacity of *A. delafieldii* to actively suppress plant immune responses in the presence of *R. irregularis*. However, *flhC*- strains, which do not express flagellin, may be less reliant on such immune evasion strategies, thereby maintaining colonization even under reduced T2SS activity. These findings highlight the complex interplay between bacterial regulatory systems, fungal signals, and plant immune responses.

This study reveals how *flhC* can broadly influence plant–fungus–bacteria interactions. Transcriptomic analyses showed that *flhC* deletion in LR140 recapitulated LR124’s expression patterns, while reintroducing *flhC* restored the original regulatory profile. We linked FlhDC to metabolism and environmental sensing, underscoring its integration in global gene regulation. Additionally, the distinct responses of *flhC+* and *flhC*− strains to root metabolome changes induced by *R. irregularis* suggest that FlhC modulates bacterial adaptation to host metabolic states. The transcriptional reprogramming following *flhC* loss included upregulation of traits advantageous for root colonization and potentially plant growth. While our reductionist approach, using defined media supplemented with root extracts, circumvented challenges associated with *in planta* transcriptomics, further validation in whole-plant and ecologically complex systems will be essential to assess the broader relevance of our findings. Future studies could integrate targeted genetic and functional approaches, including site-directed mutagenesis, DNA binding assays (e.g. by ChIP-seq), and the use of fluorescent reporter strains, to dissect the mechanistic role of FlhDC in mediating transcriptional responses to diverse biotic and abiotic cues and bacterial colonization of roots.

## Supplementary Material

ycaf235_Supplemental_Files

## Data Availability

The raw sequencing data have been deposited in the NCBI Sequence Read Archive (SRA) under the BioProject PRJNA1167168. The datasets and code supporting the conclusions of this article are available in a Zenodo repository at [99] with the URL https://doi.org/10.5281/zenodo.13836969. Code is also deposited at and available from [100] with the URL https://github.com/rsiani/acidovorax_flhc. For strains LR124 and LR140 availability, refer to [23].
