# Supplementary material for: Loss of the flagellar regulator FlhC changes the transcriptional response of plant-associated Acidovorax delafieldii strains to metabolites from Rhizophagus irregularis-colonized Lotus japonicus roots
Source: ISME Commun. 2026 Jan 19;6(1):ycaf235. doi: 10.1093/ismeco/ycaf235 (PMC12888815; doi:10.1093/ismeco/ycaf235)
Supplement: ycaf235_Supplemental_Files [file ycaf235_supplemental_files.zip › SupplementaryTable1_ycaf235.docx]

**Supplementary Table 1:** Examples of terms in the PLaBAse ontology.

| Hierarchical Level | Term | Explanation | Example Gene | Function | KEGG ID |
| --- | --- | --- | --- | --- | --- |
| PGP1 | Direct Effects | Bacterial function with potentially direct effects on plant growth | nifA\|anfA\|vnfA | Fixation of atmospheric nitrogen | K02584 |
| PGP2 | Stress Control | Participate in bacterial tolerance and resistance of stress | gst | Glutation S-transferase | K00799 |
| PGP3 | Xenobiotics biodegradation | Bacterial degradation of potentially toxic compounds | badK | Anaerobic Benzoate Degradation | K07534 |
| PGP4 | Root colonization by nodulation | Facilitate symbiosis in nodule forming rhizobacteria | nodA | Acyltransferase | K14658 |
| PGP5 | Plant Derived Putrescine Transport | Transport of plant derived metabolites | puuP | Putrescine importer | K14052 |

In the PLaBAse ontology, bacterial protein-coding gene families are leaves of a hierarchical tree organized in 7 layers (from PGP0 to PGP6) of increasing resolution. Here, example terms for layers 1 to 5 are provided, along with a representative gene family and its function.a
