# Supplementary material for: Loss of the flagellar regulator FlhC changes the transcriptional response of plant-associated Acidovorax delafieldii strains to metabolites from Rhizophagus irregularis-colonized Lotus japonicus roots
Source: ISME Commun. 2026 Jan 19;6(1):ycaf235. doi: 10.1093/ismeco/ycaf235 (PMC12888815; doi:10.1093/ismeco/ycaf235)
Supplement: ycaf235_Supplemental_Files [file ycaf235_supplemental_files.zip › SupplementaryTable2_ycaf235.docx]

**Supplementary Table 2a** Strains and plasmids used in this study

| Strains or Plasmids | Relevant characteristics | Source |
| --- | --- | --- |
| *Acidovorax* LR140 | Wild type strain | This study |
| *E. coli* strains |  |  |
| Top 10 | Host for cloning | Lab collection |
| ST18 | Host for conjugation | Lab collection |
| Plasmids |  |  |
| pK19mobsacB | Suicide cloning vector, Kan^R^ | Lab collection |
| pUC57 | Complement vector, Gent | Lab collection |

**Supplementary Table 2b** PCR primers used in this study

| Primer | Sequence (5’-3’)^a^ |
| --- | --- |
| For deletion |  |
| P1_flhC_up_F | ATGAAGACTTTACGCCATGCGGCGCATCCGAAG |
| P2_flhC_up_R | CACGCCAAGGTGGGCTTTCGGTGAGAACGGGAC |
| P3_flhC_down_F | TCACCGAAAGCCCACCTTGGCGTGTTTTTTG |
| P4_flhC_down_R | ATGAAGACTTCAGAGCACCGTGATGCGCCGTTT |
| For complement |  |
| P5_flhC_F | ATGAAGACTTTACGTCACAATCCATTTCAACGGATC |
| P6_flhC_R | ATGAAGACTTCAGATCAATGCAGCATCAGCGCG |
